# Comparing prognostic value of preoperative platelet indexes in patients with resectable gastric cancer

**DOI:** 10.1038/s41598-022-10511-6

**Published:** 2022-04-20

**Authors:** Hongtai Shi, Hongsheng Wang, Jie Pan, Zhenhua Liu, Zuoan Li

**Affiliations:** 1 Department of Radiation Oncology, Affiliated Yancheng Hospital, School of Medicine, Southeast University, Yancheng Third People’s Hospital, 75 Juchang Street, Yancheng, 224005 China; 2 Department of Radiotherapy, The Yancheng Clinical College of Xuzhou Medical University, The First People’s Hospital of Yancheng, 66 Renmin Road, Yancheng, 224005 China; 3 Department of General Surgery, The Yancheng Clinical College of Xuzhou Medical University, The First People’s Hospital of Yancheng, 66 Renmin Road, Yancheng, 224005 China; 4 Department of Oncology, Affiliated Yancheng Hospital, School of Medicine, Southeast University, Yancheng Third People’s Hospital, 75 Juchang Street, Yancheng, 224005 China; 5 Department of Interventional Radiology, Affiliated Yancheng Hospital, School of Medicine, Southeast University, Yancheng Third People’s Hospital, 75 Juchang Street, Yancheng, 224005 China

**Keywords:** Gastrointestinal cancer, Prognostic markers, Surgical oncology

## Abstract

The ratio of mean platelet volume (MPV) to count (PC) (MPV/PC) has been applied in the diagnosis and prognosis of various malignancies. However, the prognostic value of MPV/PC in gastric cancer has not been studied yet. This study aims to explore the prognostic value of neutrophil-to-lymphocyte ratio (NLR), platelet-to-lymphocyte ratio (PLR), combined neutrophil-platelet score (CNPS), systemic immune-inflammation index (SII) and MPV/PC in patients with resectable gastric cancer. In this study, the medical records of patients with gastric cancer in two centers were retrospectively analyzed. Kaplan–Meier and log-rank were tests applied to analyze the survival differences of patients with various inflammation indexes. A nomogram prognostic model was established to predict the 3- and 5-year survival rate of patients with resectable gastric cancer. In the two cohorts, Kaplan–Meier analysis that the postoperative survival time of gastric cancer patients with low MPV/PC, high NLR, high PLR and high SII was significantly shorter than that of patients with high MPV/PC, low NLR, low PLR or low SII. Compared with NLR, PLR, SII and CNPS, MPV/PC was more accurate in determining the prognosis of patients with gastric cancer than other indexes, and multivariate analysis confirmed that MPV/PC was an independent prognostic factor for patients with resectable gastric cancer. The nomogram model established based on tumor size, TNM stage and MPV/PC was more accurate than TNM stage in predicting the 3- and 5-year survival rate of patients with resectable gastric cancer. Preoperative MPV/PC is a new independent prognostic index and a potential marker for treatment response monitoring in patients with resectable gastric cancer. The nomogram model for postoperative prognosis of gastric cancer established based on MPV/PC, tumor size and TNM stage is helpful for developing more accurate and timely individualized therapeutic regimens.

## Introduction

Gastric cancer, as one of the common tumors of the digestive tumor, is the fourth most common tumor in the world, with a poor prognosis and a serious threat to human health^1^. According to statistics, there were more than 1 million new cases worldwide in 2020, and about 769,000 deaths of gastric cancer. The incidence rate of gastric cancer in North America and most Western European countries has declined, but its morbidity and mortality rates in China remain high^1^. Early gastric cancer has a good prognosis, with a 5-year survival rate of more than 95%, while advanced gastric cancer has a poor prognosis, with a 5-year survival rate of less than 20%^2^. It is currently recognized that TNM stage is a reliable predictor for the prognosis of gastric cancer. However, due to the many influencing factors for the prognosis of gastric cancer, individual difference is great among patients, and TNM stage alone may have limitations in prediction. Therefore, it is extremely necessary to find a simple, easy-to-obtain and economical tumor detection index related to the clinicopathological characteristics and prognosis of gastric cancer.

In recent years, the relation between malignancies and inflammation has become a research hotspot in the field of tumors^3^. A number of studies have revealed that inflammation-related indexes based on routine blood tests can be applied as potential prognostic factors for gastric cancer, such as neutrophil-to-lymphocyte ratio (NLR), platelet-to-lymphocyte ratio (PLR), combined neutrophil-platelet score (CNPS), and systemic immune-inflammation index (SII)^4–8^. Most of these indexes include platelet count (PC), showing the prognostic value of PC in gastric cancer. However, PC only represents the number of platelets and does not take into account the activity of platelets. Mean platelet volume (MPV) is an index of platelet function status and an index of platelet activation and turnover rate. Studies have revealed that larger platelets are more metabolically and functionally active^9^. MPV and PC are important parameters of platelets, which display significant changes in the pathophysiological process of thrombosis with the progression of disease^10^. According to the latest research, MPV/PC can be applied for the diagnosis and prognosis of certain malignancies including hepatocellular carcinoma, pancreatic cancer, lung cancer, nasopharyngeal carcinoma, glioma, colorectal cancer, and esophageal cancer^11–20^. However, there is no relevant research on this ratio for prognostic judgment of gastric cancer. This study aims to explore the prognostic value of preoperative platelet indexes (NLR, PLR, SII, CNPS, and MPV/PC) through two centers, and then compare the performance of the five in predicting the prognosis of patients after gastric cancer, so as to select the optimal assessment factors for the prognosis of patients with gastric cancer after surgery, further establish a prognostic nomogram for resectable gastric cancer, and compare it with the traditional AJCC-TNM stage, thereby determining whether the model can provide more accurate prognostic judgments for patients.

## Results

### Prognostic value of NLR, PLR, SII and MPV/PC in patients with radical gastric cancer surgery

**Figure 1:**
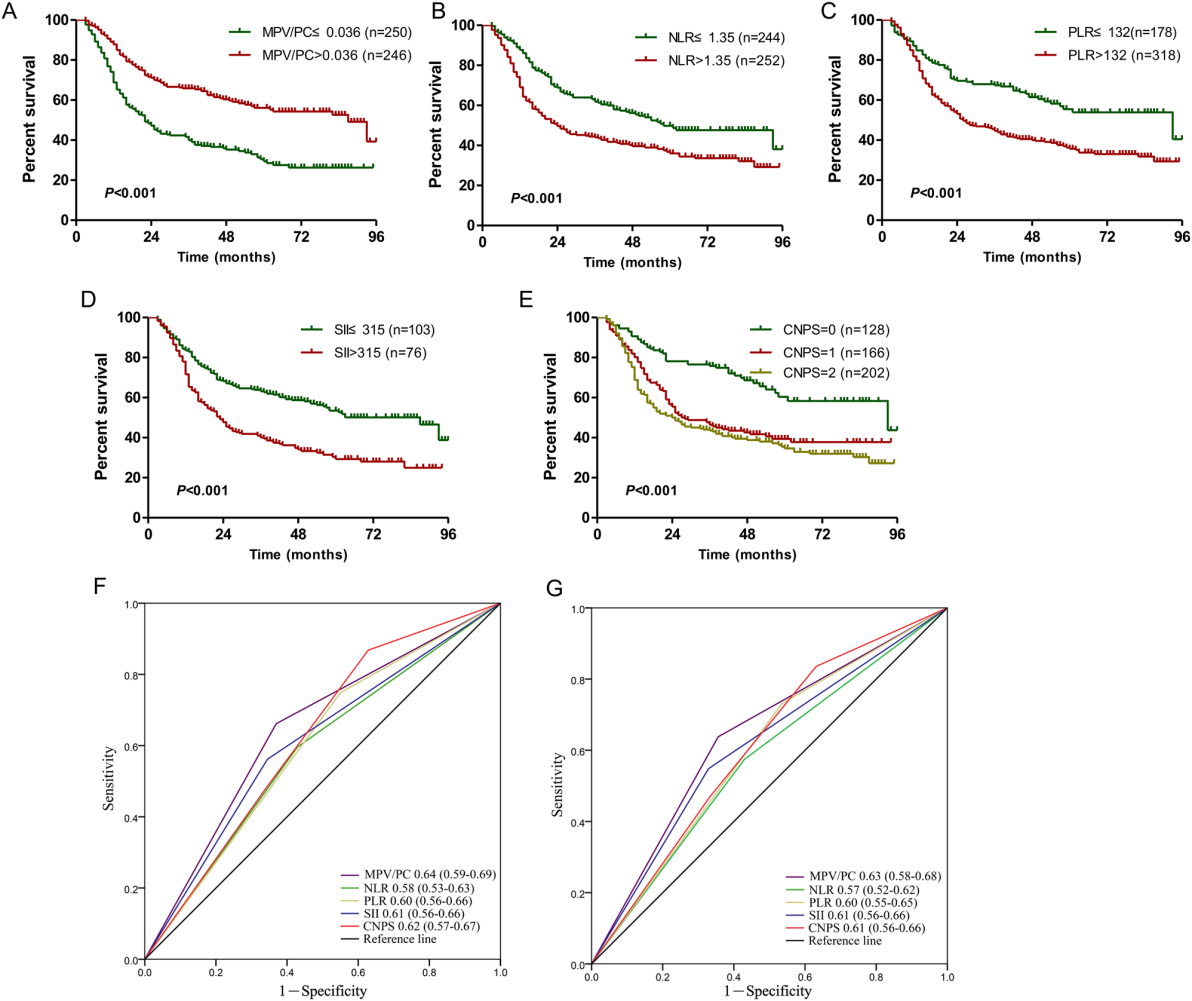
The prognostic significance of preoperative MPV/PC (**A**), NLR (**B**), PLR (**C**), SII (**D**), and CNPS (**E**) in gastric cancer in the primary cohort. Predictive ability of the MPV/PC in gastric cancer was compared with NLR, PLR, SII and CNPS by ROC curves in 3-years (**F**) and 5-years (**G**) in the primary cohort.

In primary cohort, the postoperative follow-up time of gastric cancer patients was 3–96 months, and the median follow-up time was 57 months. Among them, the median survival time of patients was 42 months (95% CI 30–54), the 3- and 5-year survival rates were 52.8% and 42.8%, respectively. According to the description in the method, NLR, PLR, SII, and MPV/PC were divided into two groups, and CNPS into three groups. Through Kaplan–Meier curve and log-rank test, it was found that the postoperative survival time of gastric cancer patients with low MPV/PC, high NLR, high PLR and high SII was significantly shorter than that of patients with high MPV/PC, low NLR, low PLR or low SII (Fig. 1A–D). The postoperative survival time of patients with a CNPS score of 0 was significantly longer than that of patients with a CNPS score of 1 or 2 points (Fig. 1E). The accuracy of each index for the prognosis of gastric cancer patients was compared by area under the curve (AUC), and it was found that the AUC of MPV/PC at 3 and 5 years was significantly larger than that of other indexes, indicating that MPV/PC has higher prognostic value for gastric cancer patients after surgery (Fig. 1F,G).

**Figure 2:**
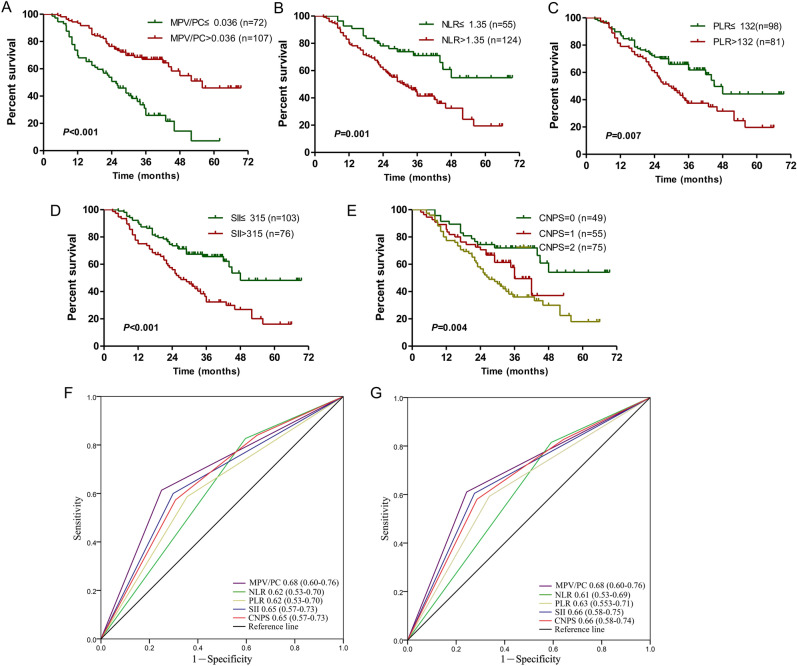
The prognostic significance of preoperative MPV/PC (**A**), NLR (**B**), PLR (**C**), SII (**D**), and CNPS (**E**) in gastric cancer in the validation cohort. Predictive ability of the MPV/PC in gastric cancer was compared with NLR, PLR, SII and CNPS by ROC curves in 3-years (**F**) and 5-years (**G**) in the validation cohort.

In validation cohort, the postoperative follow-up time of gastric cancer patients was 3–70 months, the median follow-up time was 37 months, the median survival time of patients was 42 months (95% CI 34–51), and the 3- and 5-year survival rates were 50.7% and 31.3%, respectively. In validation cohort, it was once again confirmed through Kaplan–Meier curve and log-rank test that the postoperative survival time of gastric cancer patients with low MPV/PC, high NLR, high PLR and high SII was significantly shorter than that of patients with high MPV/PC, low NLR, low PLR, or low SII (Fig. 2A–D). The postoperative survival time of gastric cancer patients with CNPS score of 1 or 2 points was significantly shorter than that of patients with CNPS score of 0 points (Fig. 2E). It was also confirmed that MPV/PC had a greater accuracy than other indexes in judging the prognosis in gastric cancer (Fig. 2F,G). Therefore, it is believed that MPV/PC is the most valuable index. Next, MPV/PC was further analyzed.

### MPV/PC was an independent prognostic factor in gastric cancer

In primary cohort, the chi-square test was applied to analyze the correlation between MPV/PC and postoperative clinicopathological factors. The results showed that patients with low MPV/PC might have larger tumor size and higher TNM stage (Table 1), which had no obvious correlation with other clinicopathological characteristics (Table 1). In addition, MPV/PC was significantly negatively correlated with other inflammation indexes (Table 1). In validation cohort, the same results were also confirmed, except that MPV/PC might be related to gender.

**Table 1:** Baseline characteristics for patients with MPV/PC > 0.036 versus MPV/PC ≤ 0.036 in primary and validation cohort.

Clinical parameter	Primary cohort				Validation cohort			
	MPV/PC > 0.036 (n = 246)	MPV/PC ≤ 0.036 (n = 250)	χ^2^	*P*	MPV/PC > 0.036 (n = 107)	MPV/PC ≤ 0.036 (n = 72)	χ^2^	*P*
**Sex**			0.63	0.427			10.23	0.001*
Male	161	172			71	63		
Female	85	78			36	9		
**Age**			3.83	0.050			2.43	0.119
≤ 60	158	181			62	50		
> 60	88	69			45	22		
**Tumor location**			0.328	0.849			2.67	0.263
Upper	45	43			16	5		
Middle	96	94			37	27		
Lower	105	113			54	40		
**Histological grade**			0.12	0.729			0.039	0.843
Well or moderately differentiated	115	113			64	42		
Poorly or not differentiated	131	137			43	30		
**Lauren type**			1.47	0.479			1.30	0.521
Diffuse	41	36			19	16		
Intestinal	106	121			49	27		
Mixed	99	93			39	29		
**Tumor size**			19.53	< 0.001*			9.43	0.002*
≤ 5	130	83			56	21		
> 5	116	167			51	51		
**Lymphovascular invasion**			0.51	0.476			0.11	0.741
No	174	184			68	44		
Yes	72	66			39	28		
**Perineural invasion**			0.88	0.348			0.18	0.671
No	148	140			76	49		
Yes	98	110			31	23		
**TNM stage (AJCC, 8th)**			35.21	< 0.001*			6.34	0.042*
I	86	32			36	12		
II	70	81			49	42		
III	90	137			22	18		
**Adjuvant chemotherapy**			2.91	0.088			0.09	0.765
No	107	90			41	26		
Yes	139	160			66	46		
**NLR**			27.113	< 0.001*			32.01	< 0.001*
NLR ≤ 1.35	150	94			50	5		
NLR > 1.35	96	156			57	67		
**PLR**			21.424	< 0.001*			12.23	< 0.001*
PLR ≤ 132	113	65			70	28		
PLR > 132	133	185			37	44		
**SII**			25.77	< 0.001*			32.30	< 0.001*
SII ≤ 315	164	110			80	23		
SII > 315	82	140			27	49		
**CNPS**			36.62	< 0.001*			29.03	< 0.001*
0	90	38			44	5		
1	83	83			32	23		
2	73	129			31	44		

*TNM* tumor, node, metastasis, *AJCC* American Joint Committee on Cancer, *MPV/PC* mean platelet volume to platelet count, *NLR* neutrophil-to-lymphocyte ratio, *PLR* platelet-to-lymphocyte ratio, *CNPS* combined neutrophil-platelet score, *SII* systemic immune-inflammation index. *Represents a statistically difference.

In primary cohort, univariate analysis showed that MPV/PC, NLR, PLR, SII and CNPS were influencing factors for the prognosis of patients with gastric cancer (Table 2). Statistically significant factors were then incorporated into the multivariate cox analysis, and it was found that tumor size, TNM stage and MPV/PC were independent prognostic factors for patients after radical gastric cancer surgery (Table 2). Validation cohort show the same results (Table 2). Therefore, it was confirmed in primary cohort and validation cohort that among MPV/PC, NLR, PLR, SII and CNPS, only MPV/PC is an independent prognostic factor for patients after radical gastric cancer.

**Table 2:** Univariate and multivariate cox regression analyses for overall survival in patients with gastric cancer.

Variables	Univariate analysis		Multivariate analysis	
	HR (95%CI)	*P* value	HR (95%CI)	*P* value
**Primary cohort**				
Sex: male vs. female	0.92 (0.71–1.18)	0.507		
Age: > 60 vs. ≤ 60	1.18 (0.91–1.53)	0.204		
Tumor location		0.002*		0.077
Middle vs. upper	0.70 (0.51–0.97)	0.029*	0.75 (0.51–1.14)	0.213
Lower vs. upper	0.57 (0.42–0.78)	< 0.001*	0.69 (0.50–0.95)	0.024*
Grade: poorly vs. well	2.16 (1.70–2.76)	< 0.001*	1.33 (0.97–1.68)	0.071
Lauren type		0.947		
Intestinal vs. diffuse	1.04 (0.74–1.48)	0.820		
Mixed vs. diffuse	1.00 (0.70–1.43)	0.997		
Tumor size: > 5 vs. ≤ 5	2.20 (1.70–2.84)	< 0.001*	1.76 (1.36–2.28)	< 0.001*
Lymphovascular: yes vs. no	1.05 (0.81–1.37)	0.703		
Perineural: yes vs. no	1.78 (1.41–2.26)	< 0.001*	1.19 (0.81–1.59)	0.173
TNM stage		< 0.001*		< 0.001*
II vs. I	2.15 (1.39–3.32)	0.001*	1.73 (1.11–2.70)	0.015*
III vs. I	5.66 (3.82–8.40)	< 0.001*	3.77 (2.48–5.73)	< 0.001*
Chemotherapy: yes vs. no	1.14 (0.90–1.45)	0.285		
MPV/PC: > 0.036 vs. ≤ 0.036	0.47 (0.37–0.60)	< 0.001*	0.61 (0.47–0.78)	< 0.001*
NLR: > 1.35 vs. ≤ 1.35	1.66 (1.30–2.11)	< 0.001*	1.25 (0.97–1.60)	0.088
PLR: > 132 vs. ≤ 132	1.83 (1.40–2.39)	< 0.001*	1.24 (0.94–1.64)	0.130
SII: > 315 vs. ≤ 315	1.89 (1.49–2.40)	< 0.001*	1.22 (0.95–1.57)	0.123
CNPS		< 0.001*		0.202
1 vs. 0	2.10 (1.47–2.99)	< 0.001*	1.39 (0.97–2.00)	0.074
2 vs. 0	2.47 (1.76–3.47)	< 0.001*	1.29 (0.89–1.86)	0.178
**Validation cohort**				
Sex: male vs. female	0.99 (0.62–1.60)	0.977		
Age: > 60 vs. ≤ 60	1.27 (1.01–1.58)	0.039		
Tumor location		0.752		
Middle vs. upper	0.91 (0.44–1.87)	0.798		
Lower vs. upper	1.09 (0.55–2.14)	0.810		
Grade: poorly vs. well	1.18 (0.96–1.45)	0.121		
Lauren type		0.794		
Intestinal vs. diffuse	1.17 (0.63–2.16)	0.625		
Mixed vs. diffuse	1.24 (0.67–2.30)	0.497		
Tumor size: > 5 vs. ≤ 5	2.22 (1.68–2.94)	< 0.001*	1.89 (1.42–2.53)	< 0.001*
Lymphovascular: yes vs. no	1.34 (0.86–2.11)	0.201		
Perineural: yes vs. no	1.28 (0.79–2.05)	0.315		
TNM stage		< 0.001*		< 0.001*
II vs. I	2.90 (1.53–5.49)	0.001*	2.21 (1.16–4.22)	0.017*
III vs. I	6.19 (3.16–12.13)	< 0.001*	4.44 (2.45–8.79)	< 0.001*
Chemotherapy: yes vs. no	1.30 (0.84–2.00)	0.240		
MPV/PC: > 0.036 vs. ≤ 0.036	0.34 (0.22–0.52)	< 0.001*	0.43 (0.28–0.67)	< 0.001*
NLR: > 1.35 vs. ≤ 1.35	2.35 (1.40–3.96)	0.001*	1.78 (0.99–3.22)	0.055
PLR: > 132 vs. ≤ 132	1.77 (1.16–2.70)	0.008*	1.62 (0.89–2.73)	0.121
SII: > 315 vs. ≤ 315	2.22 (1.45–3.38)	< 0.001*	1.56 (0.99–2.43)	0.051
CNPS		0.003*		0.076
1 vs. 0	1.75 (0.92–3.32)	0.087	1.47 (0.73–2.93)	0.278
2 vs. 0	2.59 (1.47–4.57)	0.001*	2.00 (1.07–3.75)	0.030*

*TNM* tumor, node, metastasis, *AJCC* American Joint Committee on Cancer, *MPV/PC* mean platelet volume to platelet count, *NLR* neutrophil-to-lymphocyte ratio, *PLR* platelet-to-lymphocyte ratio, *CNPS* combined neutrophil-platelet score, *SII* systemic immune-inflammation index. *Represents a statistically difference.

### Establishment and validation of prognostic nomogram after gastric cancer

**Figure 3:**
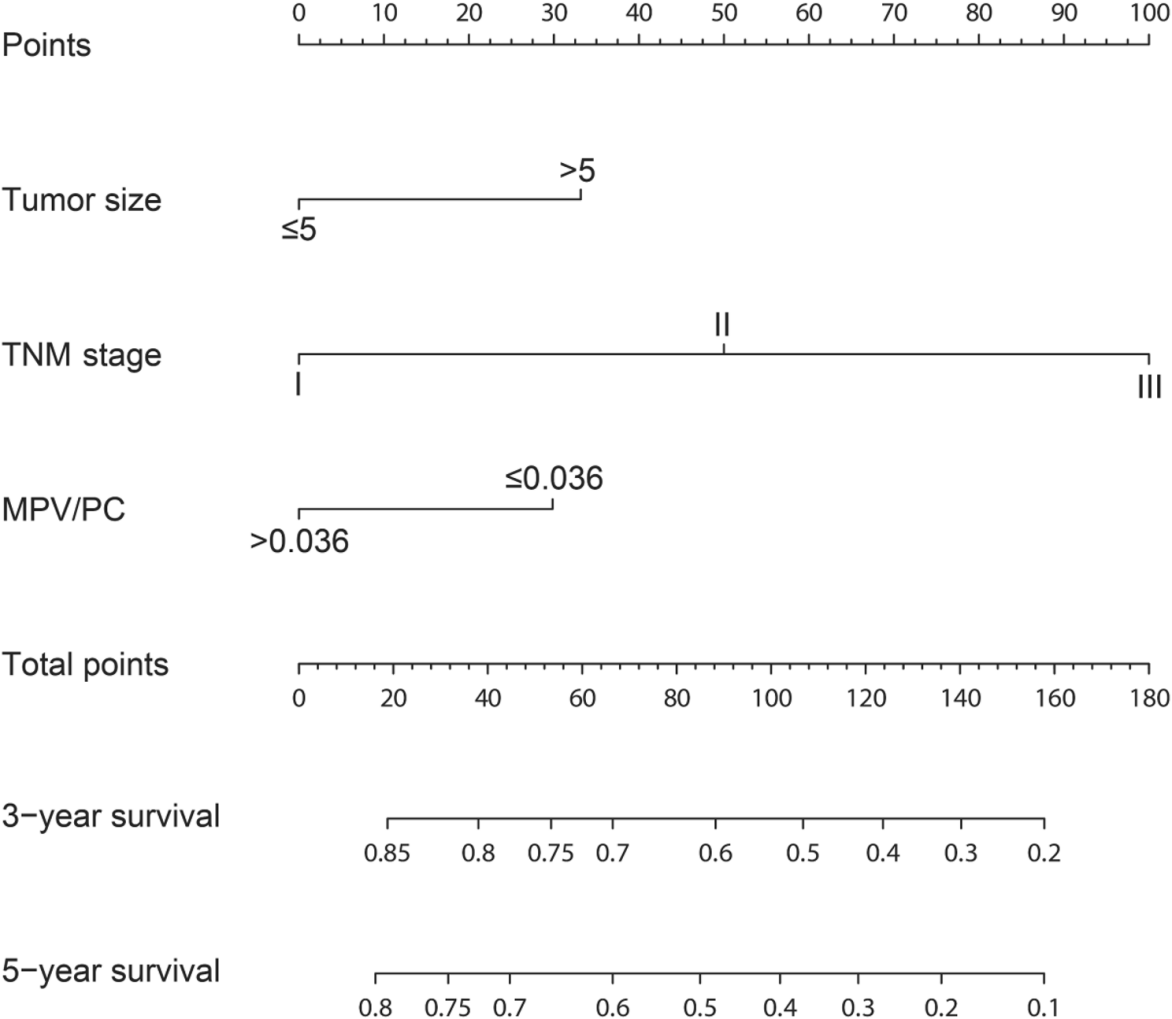
The nomogram integrating tumor size, MPV/PC and TNM stage for OS of patients with resectable gastric cancer.

**Figure 4:**
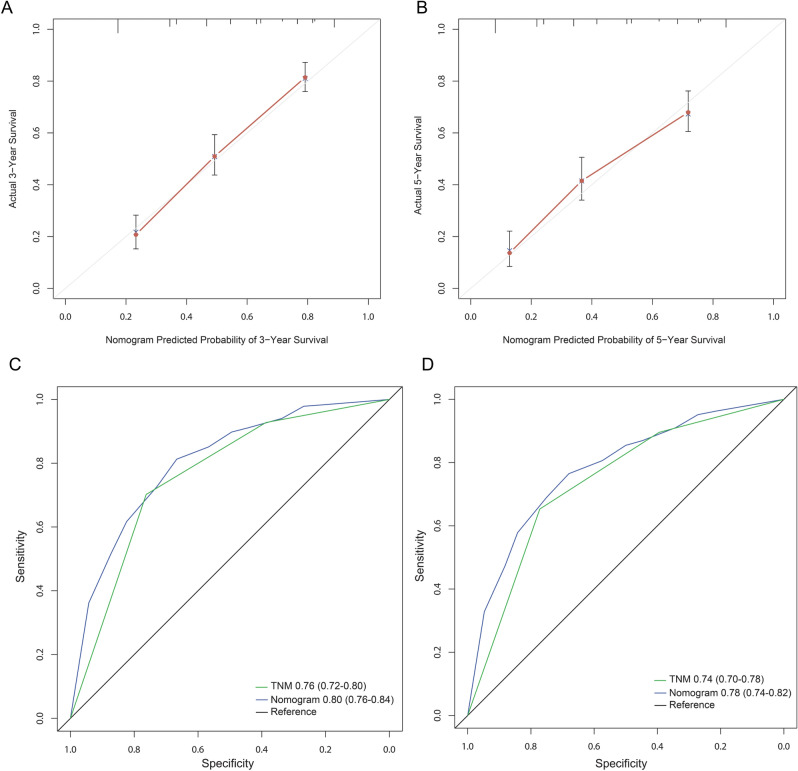
The 3-year (**A**) or 5-year (**B**) survival rate of resectable gastric cancer patients predicted by nomogram is highly consistent with the actual observed values in the primary cohort. The ability of ROC analysis nomogram to predict the 3-year (**C**) or 5-year (**D**) survival rate of resectable gastric cancer patients, the nomogram has a larger AUC than TNM staging in the primary cohort.

**Figure 5:**
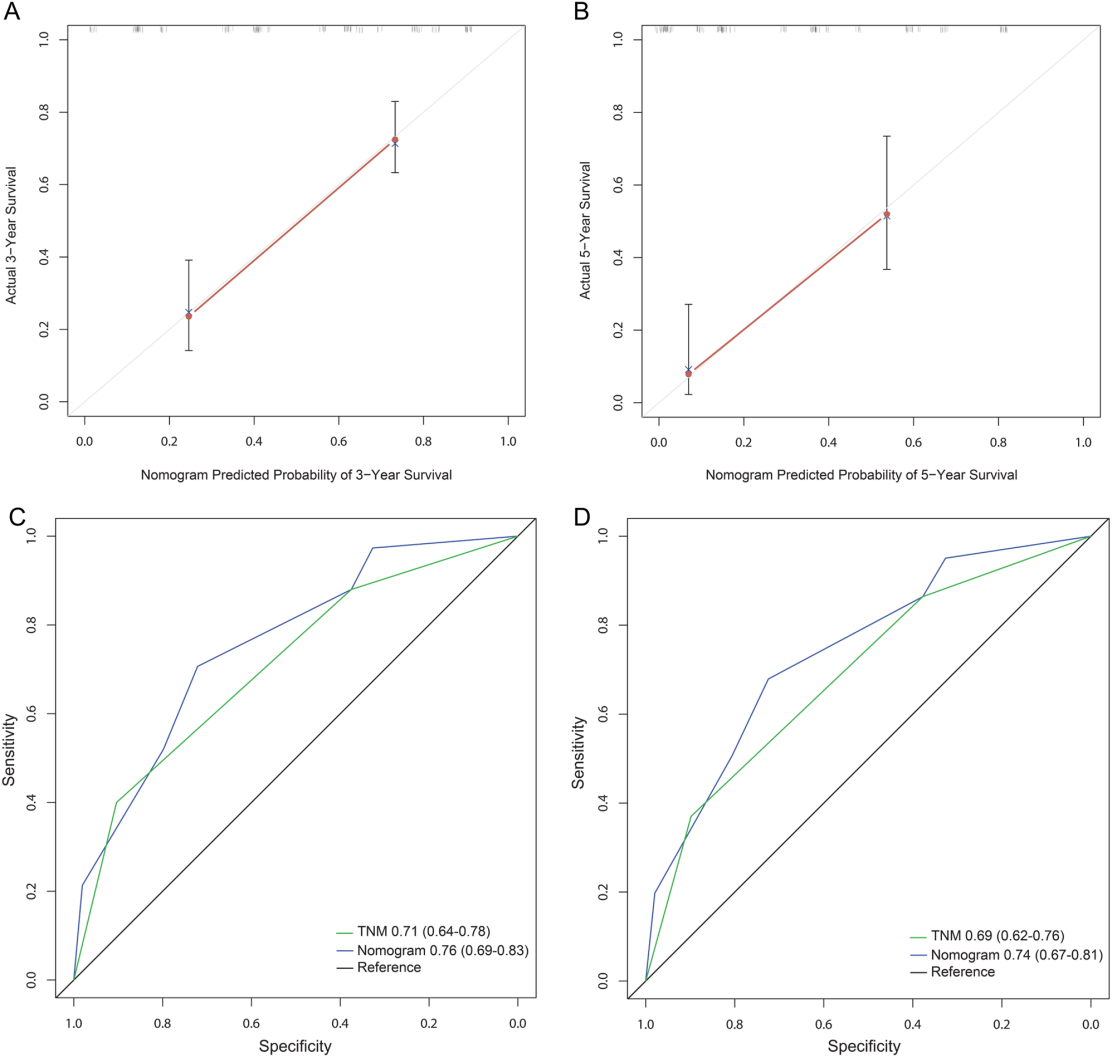
The 3-year (**A**) or 5-year (**B**) survival rate of resectable gastric cancer patients predicted by nomogram is highly consistent with the actual observed values in the validation cohort. The ability of ROC analysis nomogram to predict the 3-year (**C**) or 5-year (**D**) survival rate of resectable gastric cancer patients, the nomogram has a larger AUC than TNM staging in the validation cohort.

In primary cohort, the independent prognostic factors (tumor size, TNM stage and MPV/PC) for OS of patients with gastric cancer after surgery were selected in the multivariate analysis to establish the nomogram model (for individual cases, tumor size, TNM stage and MPV/PC corresponded to the uppermost Points, the Points added up corresponded to the lower Total Points, and then the Total Points corresponded to the lowermost 3-year and 5-year survival rate, so that it can be applied to estimate individual cases) (Fig. 3). The ability of nomogram model to predict the degree of discrimination was assessed by AUC, and the 3- or 5-year AUC of nomogram was significantly larger than that of TNM stage (Fig. 4A,B). The consistency test was performed by drawing a calibration curve between the predicted value and the actual value. The results showed that the 3- or 5-year survival rate of gastric cancer patients predicted by the nomogram had a great correlation with the actual survival rate (Fig. 4C,D), confirming that there is no significant difference between the predicted risk by the nomogram and the actual rate. Then validation cohort was used to externally validate the nomogram. In validation cohort, it was also confirmed that the 3- or 5-year AUC of the nomogram was significantly larger than that of TNM stage (Fig. 5A,B), and there was no significant difference between the predicted risk by the nomogram and the actual rate (Fig. 5C,D), confirming that the nomogram established based on tumor size, TNM stage and MPV/PC is an effective model for assessing the 3- and 5-year survival rate of gastric cancer patients after surgery.

## Discussion

Recently, domestic and foreign studies have shown that inflammation is closely related to the occurrence and development of tumors^3^. Gasic et al*.* first clarified that increased PC can promote tumor growth and metastasis^21^, and it has been confirmed in various studies that increased PC in peripheral blood is a risk factor for poor prognosis of various malignancies^22–24^. With the deepening of research, it has been found that platelet activation also plays a role in the occurrence and development of malignancies^25^. MPV is one of the assessment indexes for platelet activation. Previous studies have suggested that MPV is related to thromboembolic diseases and cardiovascular diseases, but it has been found recently that MPV is related to the prognosis of various tumors^26,27^. The latest research proves that the MPV/PC combined with platelet activity and count can better reflect the role of platelets in the diagnosis and prognosis of malignancies. Cho et al*.* reported for the first time that MPV/PC has high sensitivity and specificity in the diagnosis of hepatocellular carcinoma (HCC), and its effect is better than that of MPV alone^18^. Furthermore, it has been proved that the nomogram based on MPV/PC can accurately predict the overall survival of Asian HCC patients after hepatectomy^13^. In esophageal squamous cell carcinoma (ESCC), MPV/PC is significantly lower than that in healthy people, and the decrease in MPV/PC is significantly correlated with local progression^28^. Feng et al. showed that lower MPV/PC is closely related to the poor prognosis of ESCC patients^16^. Therefore, MPV/PC is a useful predictor of the prognosis of ESCC patients. Wu et al*.* found that MPV/PC is a promising diagnostic biomarker for distinguishing benign and malignant colorectal cancer as well as early and late colorectal cancer, which can help differential diagnosis of early and late colorectal cancer^14^. In NSCLC, low MPV/PC is an important factor in predicting the poor prognosis of patients, which is more valuable than MPV or PC alone^14,17^. In nasopharyngeal carcinoma, MPV/PC is significantly lower than that in patients with nasopharyngeal benign tumors and healthy subjects, and the low MPV/PC is also statistically different in different stages and serous invasion in nasopharyngeal carcinoma^15^. In gastric cancer, Pietrzyk applied MPV/PC in the diagnosis of gastric cancer for the first time, but MPV/PC in patients with gastric cancer and healthy subjects is comparable and has no statistically significant difference^20^. In this study, the prognostic value of MPV/PC in gastric cancer was explored for the first time. Compared with other PC-based indexes (PLR, SII, CNPS and MPV/PC), MPV/PC was an independent prognostic factor in gastric cancer, which can determine the survival of patients with gastric cancer more accurately; the lower the preoperative MPV/PC, the worse the prognosis of gastric cancer patients after surgery. In addition, a nomogram model was established based on MPV/PC, which could further improve the judgment of TNM stage on the survival of patients with gastric cancer, and provide clinicians with ideas in selecting the treatment of gastric cancer.

MPV/PC may be involved in tumorigenesis and development in several ways: (1) Platelets participate in the inflammatory response and mediate tumor angiogenesis and distant metastasis. Tumor cells promote platelet maturation by secreting cytokines. Due to stronger and faster reactivity, larger platelets are able to secrete more granular substances that mediate the inflammatory response. Moreover, platelets promote a variety of cytokines, such as platelet-derived agglutination factors, which promote tumorigenesis and metastasis^29^. Tumor and platelets are inseparably related via inflammation, influenced and cause—and—effect each other. Therefore, there is an inextricable connection between PC and tumorigenesis/progression. (2) The inflammatory factor IL-6 has been reported to induce tumorigenesis and metastasis through various signal pathways. More precisely, IL-6 not only induces the differentiation and proliferation of bone marrow megakaryocytes and early progenitor cells, but also directly acts on specific receptors on megakaryocytes^25^. Evidently, platelet activation may be a signal of tumor progression. As an indicator of platelet activation, MPV can partially reflect the state of systemic immunity, and can also be used to monitor tumor progression. Therefore, compared with MPV or PC alone, MPV/PC can better reflect the relation between platelets and tumors.

This study obtained some good results, but there were still some deficiencies. First, this study was a retrospective study, and some confounding factors failed to be completely excluded. There was certain heterogeneity in the postoperative treatment of patients, which may affect the clinical prognosis. Second, the follow-up period was relatively short, some patients did not reach the study endpoint, and there was certain censored value. In the future, therefore, the follow-up period should be extended to improve the quality of the research, and the correlation between MPV/PC and prognosis of gastric cancer should be evaluated through multi-center large-scale prospective clinical research.

## Conclusion

Preoperative peripheral blood MPV/PC is a new independent prognostic index and a potential marker for treatment response monitoring in patients with gastric cancer after surgery. The nomogram model for postoperative prognosis of gastric cancer established based on MPV/PC, tumor size and TNM stage can more objectively and reliably predict the survival of patients with gastric cancer than traditional TNM staging system, which is helpful for developing more accurate and timely individualized therapeutic regimens.

## Methods

### Patients

This study was a retrospective study and it enrolled cases (n = 496) of gastric cancer patients receiving radical surgery in The First people’s Hospital of Yancheng from 2011 to 2017 as primary cohort. In addition, gastric cancer patients (n = 179) receiving radical resection in the Yancheng Third People’s Hospital were enrolled as validation cohort. Inclusion criteria Inclusion and exclusion criteria are consistent with our previous article^30^. All patients signed the informed consent. This study adhered to the *Declaration of Helsinki*. This study was approved by the Ethics Committee of The First people’s Hospital of Yancheng and Yancheng Third People's Hospital.

### Follow-up

The patients were followed up after surgery through outpatient or hospitalization, and the survival status was clarified by telephone. The follow-up was made once every 2–4 months in the first 2 years after surgery, and then once every 6–12 months. Follow-up content included comprehensive and detailed medical history, tumor marker examination, upper gastrointestinal angiography, chest, upper abdomen CT or ultrasound and other imaging examinations. The follow-up ended in January 2020. Overall survival (OS) was defined as the number of months from the date of surgery to the date of death or the end of follow-up.

### Definition of related indexes

The blood routine data were collected at 1 week before surgery. The lymphocytes, neutrophils, monocytes, PC and MPV in the collected specimens were detected by an automatic peripheral blood analyzer. NLR, PLR, and SII were calculated according to our previous paper^8^. MPV/PC was defined as the ratio of MPV to PC. In primary cohort, NLR, PLR, SII and MPV/PC were divided into high NLR group (NLR > 1.35), high PLR group (PLR > 132), high SII group (SII > 315), high MPV/PC group (MPV/PC > 0.036), low NLR group (NLR ≤ 1.35), low PLR group (PLR ≤ 132), low SII group (SII ≤ 315) and low MPV/PC group (MPV/PC ≤ 0.036) through receiver operating characteristic (ROC) curve analysis. CNPS was defined as 0 points for NLR ≤ 1.35 and PLR ≤ 132, 1 point for NLR > 1.35 or PLR > 132, and 2 points for NLR > 1.35 and PLR > 132.

### Statistical methods

All data were statistically analyzed using SPSS22.0 software and R 3.6.2. The chi-square test was applied to analyze the correlation between various inflammatory indexes and the clinicopathological characteristics of gastric cancer patients, and then the Kaplan–Meier method was applied to analyze the survival differences of patients with various inflammatory indexes. Log-rank test was applied to compare the results of the analysis between groups, so as to find the factors related to the prognosis of gastric cancer. Then the Cox proportional hazard regression model was used for multivariate analysis to find independent risk factors. Based on the results of multivariate Cox and other proportional hazard models, independent prognostic factors were screened and a nomogram model was established to predict the 3- and 5-year survival rate. The accuracy evaluation index of the model was the AUC. The Bootstrap (n = 1000) method was used to estimate the calibration curve of the model, so as to compare the difference between the real values and the predicted values. In addition, validation cohort was used to assess the extrapolation of the prediction model. All tests were two-sided, and P < 0.05 was considered to be statistically significant.

### Ethics approval and consent to participate

The study protocol was performed in accordance with the guidelines outlined in the Declaration of Helsinki. The Ethics Committee of The First people’s Hospital of Yancheng and Yancheng Third People's Hospital approved the study, and all participants signed informed consent statements.

## Data Availability

The data used to support the findings of this study are available from the corresponding author upon request.
